# Supplementary material for: Plant mixture balances terrestrial ecosystem C:N:P stoichiometry
Source: Nat Commun. 2021 Jul 27;12:4562. doi: 10.1038/s41467-021-24889-w (PMC8316448; doi:10.1038/s41467-021-24889-w)
Supplement: Supplementary file 1 — Supplementary Figures and Tables [file 41467_2021_24889_MOESM1_ESM.pdf]

Supplementary Information

Supplementary Table 1. The effect (P values) of mean annual ecosystem type, the proportion of N-fixing plants, solar radiation, annual aridity index (AI, indicating more climate moisture availability), soil type, background soil nutrient status and management practice (fertilization or not), on natural log response ratios (ln*RRs*) of C:N:P ratios of plant, soil, soil microbial biomass and enzyme.

Attribute		Plant C:N	Plant C:P	Plant N:P	Soil C:N	Soil C:P	Soil N:P	Microbial biomass C:N	Enzymatic activity C:N	Enzymatic activity C:P	Enzymatic activity N:P
Ecosystem type	df	3,45	2,78	3,22	3,62	2,10	3,34	3,15	3,32	3,18	3,28
	F	0.41	2.67	0.42	0.17	0.84	0.51	1.11	0.15	0.19	2.21
	P	0.747	0.076	0.744	0.919	0.459	0.678	0.378	0.931	0.902	0.109
	R^2	0.006	0.028	0.013	0.004	0.025	0.01	0.017	0.003	0.005	0.052
N-fixing plants proportion	df	1,106	1,106	1,62	1,222	1,33	1,141	1,29	1,189	1,70	1,129
	F	2.49	0.93	0.44	1.01	0.005	0.03	0.15	0.02	0.02	0.12
	P	0.117	0.338	0.512	0.317	0.946	0.857	0.698	0.899	0.896	0.727
	R^2	0.009	0.003	0.002	0.004	<0.001	<0.001	<0.001	<0.001	<0.001	<0.001
Solar radiation	df	1,31	1,31	1,22	1,73	1,9	1,28	1,15	1,23	1,7	1,20
	F	0.09	0.5	0.6	0.1	3.31	0.11	0.37	0.16	0.03	0.26
	P	0.767	0.486	0.446	0.752	0.101	0.739	0.554	0.691	0.871	0.616
	R^2	0.001	0.001	0.005	0.001	0.056	0.001	0.002	0.003	<0.001	0.005
Aridity index	df	1,34	1,28	1,36	1,71	1,10	1,33	NA	1,34	1,8	1,33
	F	1.65	2.16	0.15	0.12	0.19	0.19	NA	0.01	0.19	0.002
	P	0.208	0.152	0.705	0.726	0.673	0.662	NA	0.932	0.673	0.965
	R^2	0.012	0.014	<0.001	<0.001	0.004	0.002	NA	<0.001	0.002	<0.001
Soil type	df	13,8	7,69	12,2	15,40	4,9	8,21	9,25	10,15	10,2	12,5

	F	2.72	1.17	3.4	0.39	0.33	0.13	1.37	0.28	2.96	3.28
	P	0.079	0.332	0.249	0.975	0.848	0.997	0.253	0.981	0.279	0.109
	R^2	0.156	0.05	0.113	0.039	0.073	0.01	0.071	0.043	0.111	0.158
Background soil nutrient status	df	NA	NA	1,12	NA	NA	NA	1,32	1,28	1,37	1,11
	F	NA	NA	0.36	NA	NA	NA	0.15	0.43	1.02	1.15
	P	NA	NA	0.501	NA	NA	NA	0.700	0.519	0.319	0.307
	R^2	NA	NA	0.006	NA	NA	NA	<0.001	0.003	0.010	0.019
Management practice	df	1,154	1,92	1,22	2,305	1,14	1,35	1,18	1,45	1,25	1,108
	F	1.22	1.09	2.14	0.37	0.002	0.53	0.56	0.23	0.44	1.5
	P	0.272	0.300	0.158	0.693	0.963	0.473	0.465	0.634	0.512	0.224
	R^2	0.003	0.005	0.018	0.002	<0.001	0.006	0.003	0.002	0.004	0.009

Linear mixed-effect models used Satterthwaite approximation for degrees of freedom (df). P and R^2 are the significance of the model and explained variance by the model, respectively. NAs indicate variables that have already been included in eqn. 5&7.

Supplementary Table 2. Effects of functional diversity in mixtures (FD_{is}), background soil nutrient availability (soil C:N, N:P, and C:P ratios) and aridity index on the natural log response ratios (ln*RRs*) of the C:N:P ratios of plant, soil, soil microbial biomass and enzyme.

Source	Estimate	df	F	*P*	R^2	VIF
**Plant C:N ($R^2_{marginal}$ = 0.033, $R^2_{conditional}$= 0.181)**						
FD_{is}	0.027	1,118	0.38	0.538	0.001	1.02
log(Background soil C:N)	-0.174	1,36	9.62	**0.004**	0.032	1.02
**Plant C:P ($R^2_{marginal}$ = 0.233, $R^2_{conditional}$= 0.277)**						
FD_{is}	-0.058	1,26	0.64	0.431	0.015	1.67
Background soil C:P	-0.004	1,17	9.45	**0.007**	0.218	1.67
**Plant N:P ($R^2_{marginal}$ < 0.001, $R^2_{conditional}$= 0.189)**						
FD_{is}	0.014	1,77	0.10	0.758	< 0.001	
**Soil C:N ($R^2_{marginal}$ = 0.090, $R^2_{conditional}$= 0.514)**						
FD_{is}	-0.012	1,384	0.22	0.639	< 0.001	1.01
Background soil C:N	-0.010	1,221	52.40	**<0.001**	0.090	1.01
**Soil C:P ($R^2_{marginal}$ = 0.197, $R^2_{conditional}$= 0.626)**						
FD_{is}	0.158	1,43	1.85	0.181	0.019	1.00
Background soil C:P	-0.002	1,56	17.73	**<0.001**	0.178	1.00
**Soil N:P ($R^2_{marginal}$ = 0.119, $R^2_{conditional}$= 0.341)**						
FD_{is}	0.037	1,126	0.33	0.564	0.002	1.00
Background soil N:P	-0.033	1,67	18.81	**<0.001**	0.117	1.00
**Microbial biomass C:N ($R^2_{marginal}$ = 0.045, $R^2_{conditional}$= 0.273)**						
FD_{is}	-0.256	1,125	6.45	**0.012**	0.020	1.41
AI	-0.309	1,112	8.13	**0.005**	0.025	1.41
**Enzyme C:N ($R^2_{marginal}$ = 0.011, $R^2_{conditional}$= 0.263)**						
FD_{is}	0.144	1,214	3.91	**0.049**	0.011	
**Enzyme C:P ($R^2_{marginal}$ = 0.022, $R^2_{conditional}$= 0.173)**						
log(FD_{is})	0.116	1,93	5.36	**0.023**	0.022	
**Enzyme N:P ($R^2_{marginal}$ < 0.001, $R^2_{conditional}$= 0.283)**						
FD_{is}	0.014	1,190	0.05	0.825	<0.001	

Note: Linear mixed-effects model fit tests used Satterthwaite approximations for denominator degrees of freedom (df). *P* and R^2 are the significance of the model and explained variance by the model, respectively.

Supplementary Table 3. Sources of original studies that examined the effects of plant species mixture on terrestrial C:N:P ratios.

**Publication**	Variable	Richness levels	Stand age (years)
**Cropland**			
Akhtar, M. *et al.* Impact of chickpea and wheat co-cropping on crop growth, nutrient uptake and root induced biochemical changes. *Soil Environ.* **32**, 152-157 (2013).	Plant N:P	2	0.13
Bukovsky-Reyes, S., Isaac, M. E. & Blesh, J. Effects of intercropping and soil properties on root functional traits of cover crops. *Agr. Ecosyst. Environ.* **285** (2019).	Plant C:N	2	1.8
Cong, W. *On Soil Organic Matter Dynamics in Species-Diverse Grasslands and Intercrop Systems* Doctor thesis, Wageningen University, (2014).	Plant C:N, Soil C:N	2	7
Coser, T. R. *et al.* Soil microbiological properties and available nitrogen for corn in monoculture and intercropped with forage. *Pesqui. Agropecu. Bras.* **51**, 1660-1667 (2016).	Soil C:N, MBC/MBN	2	3.25
Du, C. *Effects of Potato/Maize Intercropping on Soil and Crops* Master thesis, Ningxia University, (2017).	Enzymatic N:P	2	2
Dyer, L., Oelbermann, M. & Echarte, L. Soil carbon dioxide and nitrous oxide emissions during the growing season from temperate maize-soybean intercrops. *J. Plant Nutr. Soil Sc.* **175**, 394-400 (2012).	Soil C:N	2	2
Fan, Z. *Mechanism of C, N coupling for improving N-fertilizer use rate inhigh density planted maize/pea intercropping system* Doctor thesis, Gansu Agricultural University (2015).	Plant C:N, Soil C:N	2	0.2
Kaci, G. *et al.* The effect of intercropping on the efficiency of faba bean - rhizobial symbiosis and durum wheat soil-nitrogen acquisition in a Mediterranean agroecosystem. *Plant Soil Environ.* **64**, 138-146 (2018).	Soil C:N	2	1
Li, D. *Soil Microbial Diversity and Interspecific Facilitation in Intercropping Between Wheat and Alfalfa* PhD thesis, Northeast Forestry University, (2015).	Enzymatic C:N, C:P, N:P	2	0.17
Li, X. *Effects of Continuous Intercropping on Crop Productivity. Stability and Soil Fertility* PhD thesis, China Agricultural University, (2017).	Enzymatic C:N, C:P, N:P	2	3, 4, 5
Lin, F., Liu, X. & Zhang, J. Study on The Contents of Carbon,Nitrogen and Enzymes Activities of Sandy Soil Grown Alfalfa and Perennial Ryegrass With Different Planting Patterns. *Grassland and Turf* 39, 43-49 (2019).	Soil C:N, Enzymatic C:N	2	6
Ma, N. *Effects of Mixed Sowing and Application of Rapeseed and Oat on Forage Yield and Soil Characters in Saline-alkali Soil* Master thesis, Inner Mongolia Agricultural University, (2019).	Enzymatic C:N	2	0.5, 1.5
Mo, J. *Soil Enzyme Activities and Nutrient Contents in Camellia oleifera-Arachis hypogaea Intercropping System*	Soil C:N, C:P, N:P, Enzymatic	2	0.25

Master thesis, Central South University of Forestry & Technology, (2017).	C:N, C:P, N:P		
Oelbermann, M. & Echarte, L. Evaluating soil carbon and nitrogen dynamics in recently established maize-soyabean inter-cropping systems. *Eur. J. Soil Sci.* **62**, 35-41 (2011).	Plant C:N, Soil C:N, MBC/MBN	2	0.5
Oelbermann, M., Regehr, A. & Echarte, L. Changes in soil characteristics after six seasons of cereal–legume intercropping in the Southern Pampa. *Geoderma Reg.* **4**, 100-107 (2015).	Soil C:N, MBC/MBN	2	1, 5
Qiao, Y. et al. Effects of Oat-legume Intercropping on Soil Enzyme Activities and Abundance of Soil Microbe. *Journal of Gansu Agricultural University* 55, 54 - 61 (2020).	Enzymatic C:N, C:P, N:P	2	1
Raza, M. A. *et al.* Effect of planting patterns on yield, nutrient accumulation and distribution in maize and soybean under relay intercropping systems. *Sci. Rep.* **9**, 4947 (2019).	Plant N:P	2	0.3, 1.3
Regehr, A., Oelbermann, M., Videla, C. & Echarte, L. Gross nitrogen mineralization and immobilization in temperate maize-soybean intercrops. *Plant Soil* **391**, 353-365 (2015).	Soil C:N	2	1, 5
Sainju, U. M., Whitehead, W. F. & Singh, B. P. Biculture Legume–Cereal Cover Crops for Enhanced Biomass Yield and Carbon and Nitrogen. *Agron J* **97**, 1403-1412 (2005).	Plant C:N	2	0.5, 1.5, 2.5
Scalise, A. *et al.* Legume-barley intercropping stimulates soil N supply and crop yield in the succeeding durum wheat in a rotation under rainfed conditions. *Soil Biol Biochem* **89**, 150-161 (2015).	Soil C:N, MBC/MBN	2	1, 2
Schoebitz, M., Castillo, D., Jorquera, M. & Roldan, A. Responses of Microbiological Soil Properties to Intercropping at Different Planting Densities in an Acidic Andisol. Agronomy 10, 781 (2020).	Plant N:P, Enzymatic C:N, C:P, N:P	2	0.5
Shang, H. *The effect of reduced fertilizer and intercropping on crop carbon and nitrogen storage and soil carbon and nitrogen fixing* Master thesis, Northwest A&F University (2014).	Plant C:N, Soil C:N	2	0.25
Sharma, R. C. & Banik, P. Baby Corn-Legumes Intercropping Systems: I. Yields, Resource Utilization Efficiency, and Soil Health. *AGROECOL. SUST. FOOD* **39**, 41-61 (2015).	Soil N:P, Enzymatic N:P	2	2
Song, Y. N. *et al.* Effect of intercropping on crop yield and chemical and microbiological properties in rhizosphere of wheat (Triticum aestivum L.), maize (Zea mays L.), and faba bean (Vicia faba L.). *Biol. Fertil. Soils* **43**, 565-574 (2007).	MBC/MBN	2	1, 2
Su, B. *Mechanism on Competition and Complementation in Different Intercropping Patterns for Cassava and Peanut* Master thesis, Hainan University, (2017).	Enzymatic C:N, C:P, N:P	2	0.3
Sun, M. M. *et al.* In situ phytoremediation of PAH-contaminated soil by intercropping alfalfa (Medicago sativa L.) with tall fescue (Festuca arundinacea Schreb.) and associated soil microbial activity. *J. Soil Sediment* **11**, 980-989 (2011).	MBC/MBN	2	0.58

Tang, X. et al. Correlation and principal component analysis of the soil environmental factors in corn/peanut intercropping system. *Ecology and Environmental Sciences* 29, 223-230 (2020).	Soil N:P, Enzymatic C:N, C:P, N:P	2	0.34
Tortorella, D. *et al.* Chemical and biological responses in a Mediterranean sandy clay loam soil under grain legume-barley intercropping. *Agrochimica* **57**, 1-21 (2013).	Plant N:P	2	0.33
Vachon, K. *Soil carbon and nitrogen dynamics and greenhouse gas mitigation in intercrop agroecosystems in Balcarce, Argentina* Master thesis, University of Waterloo (2008).	Plant C:N, Soil C:N	2	1
Wang, J. *Effects of Corn/Alfalfa Intercropping on Soil Nutrient, Enzyme Activity and Plant Growth Master thesis, Guizhou University, (2019).*	Soil N:P, Enzymatic C:N, C:P, N:P	2	1
Wang, Z. G. et al. Intercropping Enhances Productivity and Maintains the Most Soil Fertility Properties Relative to Sole Cropping. *Plos One* 9 (2014).	Enzymatic N:P	2	2, 3
Wang, Z.-g. et al. Intercropping maintains soil fertility in terms of chemical properties and enzyme activities on a timescale of one decade. Plant Soil 391, 265-282 (2015).	Enzymatic C:N, C:P, N:P	2	2, 3
Xu, Y. B., Qiu, W. W., Sun, J. P., Muller, C. & Lei, B. K. Effects of wheat/faba bean intercropping on soil nitrogen transformation processes. *J. Soil Sediment* **19**, 1724-1734 (2019).	Soil C:N	2	1
**Forest**			
Ahmed, I. U. *et al.* Polyculture affects biomass production of component species but not total standing biomass and soil carbon stocks in a temperate forest plantation. *Ann. Forest Sci.* **76**, 91 (2019).	Soil C:N	3	4
Alberti, G. *et al.* Tree functional diversity influences belowground ecosystem functioning. *Appl. Soil Ecol.* **120**, 160-168 (2017).	Soil C:N, Enzymatic C:N, C:P, N:P	3	12
Balieiro, F. C. *et al.* Accumulation and distribution of aboveground biomass and nutrients in pure and mixed stands of guachapele and eucalyptus. *J. Plant Nutr.* **25**, 2639-2654 (2002).	Plant N:P	2	5
Baum, C. et al. Mixture of Salix Genotypes Promotes Root Colonization With Dark Septate Endophytes and Changes P Cycling in the Mycorrhizosphere. *Front Microbiol* 9, (2018).	Enzymatic C:P	2	2, 3
Bini, D. *et al.* Eucalyptus grandis and Acacia mangium in monoculture and intercropped plantations: Evolution of soil and litter microbial and chemical attributes during early stages of plant development. *Appl. Soil Ecol.* **63**, 57-66 (2013).	Soil C:N, MBC/MBN	2	1
Bini, D., dos Santos, C. A., da Silva, M. C. P., Bonfim, J. A. & Cardoso, E. J. B. N. Intercropping Acacia mangium stimulates AMF colonization and soil phosphatase activity in Eucalyptus grandis. *Sci Agr* 75, 102-110 (2018).	Enzymatic C:P	2	1

Cao, B. & Wu, L. Studies on Soil Enzyme Activity and Soil Nutrient Content of Mixed Stands with Robinia pseudoacacia and Fraxinus velutina in Coastal Saline Soil. *Journal of Soil and Water Conservation* 22, 128-133 (2008).	Enzymatic C:N	2	25
Casanova-Lugo, F. *et al.* Forage yield and quality of Leucaena leucocephala and Guazuma ulmifolia in mixed and pure fodder banks systems in Yucatan, Mexico. *Agroforest Syst.* **88**, 29-39 (2014).	Plant C:N	2	4
Chen, J. & Yang, N. Effects of five plantations on soil properties in subtropical red soil hilly region. *Journal of J. For. Res.* **41**, 168-173 (2013).	Soil C:N, C:P, N:P, MBC/MBN, Enzymatic C:N	2	15
Chen, Y.-l. *et al.* Nutrient characteristics in rhizosphere of pure and mixed plantations of Manchurian walnut and Dahurian larch. *J. For. Res.* **12**, 18-20 (2001).	Soil N:P	2	11
Chodak, M. & Niklinska, M. Effect of texture and tree species on microbial properties of mine soils. *Appl. Soil Ecol.* **46**, 268-275 (2010).	Soil C:N, Enzymatic N:P	2	24
Chodak, M. & Niklinska, M. The effect of different tree species on the chemical and microbial properties of reclaimed mine soils. *Biol. Fertil. Soils* **46**, 555-566 (2010).	Soil C:N, Enzymatic N:P	2	23
Dillen, M. *et al.* How does neighbourhood tree species composition affect growth characteristics of oak saplings? *Forest Ecol. Manag.* **401**, 177-186 (2017).	Plant C:N	2, 3, 4	5
Domisch, T. *et al.* Does species richness affect fine root biomass and production in young forest plantations? *Oecologia* **177**, 581-594 (2015).	Soil C:N	2, 3, 4, 5	8, 14
Dong, H. *Soil Characteristics and Improvement of Long-term Plantation in the Yellow River Delta* Master thesis, Shan Dong Agricultural University (2014).	Soil C:N	2	10
Dong, M. *et al.* Soil microbial biomass C, N and diversity characteristics in pure and mixed forest of *Pinus* and *Cinnamomun*. *Journal of Central South University of Forestry & Technology* **37**, 146-153 (2017).	Soil C:N, MBC/MBN	2	27
Duo, Y. *et al.* The Biomass Comparison of Soil Microbial Carbon and Nitrogen of 3 Kinds of Forest Types in Subtropics. *Chinese Agricultural Science Bulletin* **28**, 14-19 (2012).	Soil C:N, MBC/MBN	2	24
Fan, S. & Yang, N. Comparison of Soil Microbiology Characteristics in Five Subtropical Ecosystems. *Journal of Tropical and Subtropical Botany* **24**, 635-641 (2016).	Soil C:N, MBC/MBN	2	15
Forrester, D. I. *Mixed-species plantations of nitrogen-fixing and non-nitrogen-fixing trees* Doctor thesis, The Australian National University (2004).	Plant N:P, Soil N:P	2	2
Forrester, D. I. *et al.* Nutrient cycling in a mixed-species plantation of Eucalyptus globulus and Acacia mearnsii. *Can. J. For. Res.* **35**, 2942-2950 (2005).	Plant C:N	2	2, 10
Forrester, D. I. *et al.* Soil Organic Carbon is Increased in Mixed-Species Plantations of Eucalyptus and Nitrogen-Fixing Acacia. *Ecosystems* **16**, 123-132 (2013).	Soil C:N, C:P, N:P	2	8.3

Gao, C. *et al.* Nutrient accumulation and cycling in pure and mixed plantations of Azadirachta indica and Acacia auriculiformis in a dry-hot valley, Yunnan Province, southwest China. *Chinese Journal of Applied Ecology* **25**, 1889-1897.	Plant N:P, Soil N:P	2	10
Garau, G. *et al.* Effect of monospecific and mixed Mediterranean tree plantations on soil microbial community and biochemical functioning. *Appl. Soil Ecol.* **140**, 78-88 (2019).	Soil C:N	2	2
Gong, X. *et al.* Sub-tropic degraded red soil restoration: Is soil organic carbon build-up limited by nutrients supply. *Forest Ecol Manag* **300**, 77-87 (2013).	Soil C:N, C:P, N:P	2	18
Guerrero-Ramírez, N. R. *et al.* Root quality and decomposition environment, but not tree species richness, drive root decomposition in tropical forests. *Plant Soil* **404**, 125-139 (2016).	Soil C:N	5	4
Guidetti Zagatto, M. R. *et al.* Acacia mangium increases the mesofauna density and diversity in the litter layer in Eucalyptus grandis plantations. *Eur. J. Soil Sci.* **94**, 103100 (2019).	Soil C:N	2	6
Gunina, A. *et al.* Response of soil microbial community to afforestation with pure and mixed species. *Plant Soil* **412**, 357-368 (2017).	Soil C:N	2, 3	10
Guo, D. *et al.* Characteristics and Evaluation of Soil Nutrients of *Pinus massoniana*, *Eucalyptus* and Mixed Plantations. *Eucalypt Science & Technology* **32**, 14 - 20 (2015).	Soil N:P	2	4
Han, Y. *Growth Rhythm of Mixed Plantation and It's Soil Properties in the Yellow River Delta* Master thesis, ShanDong Agriculture University, (2015).	Soil N:P, Enzymatic C:N, C:P, N:P	2	29
Heling, X. *Investigation on root characteristics and functions for typical sand-fixation plantations in Gonghe basin, Qinghai* PhD thesis, Chinese Academy of Forestry (2018).	Plant C:N, C:P, N:P, Soil C:N, C:P, N:P	2	9, 25
Hu, B. *et al.* Comparison of nitrogen nutrition and soil carbon status of afforested stands established in degraded soil of the Loess Plateau, China. *Forest Ecol. Manag.* **389**, 46-58 (2017).	Plant C:N, Soil C:N, MBC/MBN	2	40
Huang, Y. *Studies on Soil quality under pure Chinese fir stand, Mixtures of Chinese fir-Broadleaves and Pure Broadleaved Stand Ecosystems* Doctor thesis, Chinese Academy of Sciences (2004).	Plant C:N, Soil C:N, N:P, MBC/MBN, Enzymatic N:P	2	20
Jiang, Y. M. *et al.* Soil soluble organic carbon and nitrogen pools under mono- and mixed species forest ecosystems in subtropical China. *J. Soil Sediment* **10**, 1071-1081 (2010).	Soil C:N, MBC/MBN	2, 3	17
Kaye, J. P. *et al.* Nutrient and carbon dynamics in a replacement series of Eucalyptus and Albizia trees. *Ecology* **81**, 3267-3273 (2000).	Soil C:N	2	16

Khlifa, R. *Effets de la diversité des arbres sur le fonctionnement de l'écosystème dans deux plantations de forêts tempérées* Doctor thesis, Université Laval Québec Canada (2016).	Plant C:N, Soil C:N	2, 4	4.5
Koutika, L.-S. *et al.* Soil P availability under eucalypt and acacia on Ferralic Arenosols, republic of the Congo. *Geoderma Reg.* **7**, 153-158 (2016).	Plant N:P	2	8
Lemma, B. Soil chemical properties and nutritional status of trees in pure and mixed-species stands in south Ethiopia. *J Plant Nutr. Soil Sc.* **175**, 769-774 (2012).	Plant N:P, Soil C:N	2	24
Li, S. *Effects of mixed model on leaf and root functional traits of Phoebe bournei and Cunninghamia lanceolata (Chinese version with English abstract)* Master thesis, Central South University of Forestry and Technology (2019).	Plant C:N, C:P, N:P, Soil C:N, C:P, N:P	2	10
Lin, L. *Study on Growth Characteristics and Nutrient Competition of Phoebe bournei - Schima superba Mixed Forest* Master thesis, Central South University of Forestry and Technology (2019).	Plant C:N, C:P, N:P, Soil C:N, C:P, N:P	2	14
Liu, J. *et al.* Effects of tree species and soil properties on the composition and diversity of the soil bacterial community following afforestation. *Forest Ecol. Manag.* **427**, 342-349 (2018).	Soil C:N, N:P, C:P	2	24
Liu, J. A *Preliminary Study on The Mixing of Magnoliaceas Glance, Michelia Hedyosperma and Eucalyptus Robusta in Three and A Half Years* Master thesis, Guangxi University, (2018).	Soil N:P, Enzymatic C:N, C:P, N:P	2	3
Liu, M. Q. *et al.* Seasonal Dynamics Of Soil Microbial Biomass And Its Significance To Indicate Soil Quality Under Different Vegetations Restored On Degraded Red Soils. *Acta Pedologica Sinica* **40**, 937-944 (2003).	Soil C:N, C:P, N:P, MBC/MBN	2	10
Liu, Y. *Soil Enzyme Activities of Different Shelterbelt Types in Saline Land of the Yellow River Delta* Master thesis, ShanDong Agriculture University, (2013).	Soil N:P, Enzymatic N:P	2	26
Luo, D. *Characteristics of carbon and nitrogen in monoculture and mixed young stands of Erythrophleum fordii and Pinus massoniana in southern subtropical China* Doctor thesis, Chinese Academy of Forestry (2014).	Plant C:N, Soil C:N	2	7
Maxwell, T. L. et al. Effect of a tree mixture and water availability on soil nutrients and extracellular enzyme activities along the soil profile in an experimental forest. *Soil Biol Biochem* 148 (2020).	Enzymatic C:N, C:P, N:P	2	10
Montagnini, F. Accumulation in aboveground biomass and soil storage of mineral nutrients in pure and mixed plantations in a humid tropical lowland. *Forest Ecol. Manag.* **134**, 257-270 (2000).	Plant N:P	4	4
Nunes, L. *et al.* Growth, soil properties and foliage chemical analysis comparison between pure and mixed stands of Castanea saliva Mill. and Pseudotsuga menziesii (Mirb.) Franco, in Northern Portugal. *Forest Syst.* **20**, 496-507 (2011).	Plant C:N, C:P, N:P	2	28

Pan, H. *Soil carbon and nitrogen transformation process research of relationship between soil microbial community structure of Michelia macclurei, Pinus massoniana plantation* Master thesis, Guangxi University (2015).	Soil C:N	2	31
Pereira, A. P. A. *et al.* Mixed Eucalyptus plantations induce changes in microbial communities and increase biological functions in the soil and litter layers. *Forest Ecol. Manag.* **433**, 332-342 (2019).	Soil C:N, Enzymatic C:N	2	3.25
Pereira, E. L. *et al.* Microbial biomass and N mineralization in mixed plantations of broadleaves and nitrogen-fixing species. *Forest Syst.* **20**, 516-524 (2011).	Soil C:N, MBC/MBN	2	11
Purahong, W. et al. Tree species, tree genotypes and tree genotypic diversity levels affect microbe-mediated soil ecosystem functions in a subtropical forest. *Sci Rep*-Uk 6, (2016).	Enzymatic C:N, C:P, N:P	4	5
Qin, J. *Study on growth dynamics and ecological function of Robinia pseudoacacia andUlmus pumila mixed forest in the Loess Plateau (in Chinese with English abstract)* Doctor thesis, Northwest A & F University (2009).	Plant C:N, C:P, N:P, Soil N:P	2	1.5
Rachid, C. T. C. C. *et al.* Mixed plantations can promote microbial integration and soil nitrate increases with changes in the N cycling genes. *Soil Biol. Biochem.* **66**, 146-153 (2013).	Soil C:N	2	2, 3
Ribbons , R. R. *Seeing the forest for the trees:Tree species effects on soil microbial communities and nutrient cycling dynamics* Doctor thesis, Bangor University (2017).	Plant C:N, Soil C:N	2	2.17
Rivest, D. *et al.* Tree communities rapidly alter soil microbial resistance and resilience to drought. *Funct. Ecol.* **29**, 570-578 (2015).	Plant N:P, Soil C:N	2	4
Salahuddin *et al.* Root order-based traits of Manchurian walnut & larch and their plasticity under interspecific competition. *Sci. Rep.* **8** (2018).	Plant C:N, Soil N:P	2	30
Salahuddin *et al.* Root Morphology and Chemistry in Manchurian Ash (*Fraxinus Mandshurica*) and Larch (*Larix Gmelinii Rupr*.) Are Dependent on Species, Root Order and Competition. *J. Anim. Plant Sci.* **30**, 115-125 (2020).	Plant C:N	2	30
Shao, D. et al. The Study on Soil Enzyme Activity in the Pinus tabulaeformis and Ostryopsis davidiana Rhizosphere in Daqingshan Mountain, InnerMongolia. Journal of Arid *Land Resources and Environment* 22, 190 - 193 (2008).	Enzymatic C:N, C:P, N:P	2	20
Stanley, W. G. & Montagnini, F. Biomass and nutrient accumulation in pure and mixed plantations of indigenous tree species grown on poor soils in the humid tropics of Costa Rica. *Forest Ecol. Manag.* **113**, 91-103 (1999).	Plant N:P	4	4
Su, X. Studies of the Productivity and Ecological Characteristics of the Mixed Forest of Michelia fujianensis and Cunninghamia lanceolata at Its Young Stage. *Journal of Cent Ral South Forestry University* 20, 76-80.	Soil N:P, Enzymatic C:N, C:P, N:P	2	6
Tan, G. *et al.* Content and Seasonal Change of Soil Labile Organic Carbon under Four Different Plantations in	Soil C:N, C:P, N:P	2	21

Degraded Red Soil Region. *Acta Agriculturae Universitatis Jiangxiensis* **36**, 434-440 (2014).			
Tan, L. *et al.* Comparison of Soil Physical and Chemical Properties of Pure Castanopsis hystrix, Pure Pinus massoniana and Mixed-species Tree Plantation in South Subtropical Area. *Journal of West China Forestry Science* **43**, 35-40 (2014).	Soil C:N, C:P, N:P	2	28
Tang, G. *et al.* Accelerated nutrient cycling via leaf litter, and not root interaction, increases growth of Eucalyptus in mixed-species plantations with Leucaena. *Forest Ecol. Manag.* **310**, 45-53 (2013).	Soil C:N, C:P, N:P, MBC/MBN	2	10
Tchichelle, S. V. *et al.* Differences in nitrogen cycling and soil mineralisation between a eucalypt plantation and a mixed eucalypt and Acacia mangium plantation on a sandy tropical soil. *South Forests* **79**, 1-8 (2017).	Soil C:N	2	7
Voigtlaender, M. *et al.* Introducing Acacia mangium trees in Eucalyptus grandis plantations: consequences for soil organic matter stocks and nitrogen mineralization. *Plant Soil* **352**, 99-111 (2012).	Soil C:N	2	6
Wang, H. *et al.* C and N stocks under three plantation forest ecosystems of Chinese fir, Michelia macclurei and their mixture. *Frontiers of Forestry in China* **2**, 251-259 (2007).	Soil C:N, Plant C:N	2	23
Wang, H. *et al.* Effects of tree species mixture on soil organic carbon stocks and greenhouse gas fluxes in subtropical plantations in China. *Forest Ecol. Manag.* **300**, 4-13 (2013).	Soil C:N	2	25
Wang, H. *et al.* Mixed-species plantation with Pinus massoniana and Castanopsis hystrix accelerates C loss in recalcitrant coniferous litter but slows C loss in labile broadleaf litter in southern China. *Forest Ecol. Manag.* **422**, 207-213 (2018).	Soil C:N	2	26
Wang, Q. Study on the growth effect of mixed forest with Cunninghamia lanceolatea, Pinus massoniana and Schima superba in mountanious region of south Fujian. *Journal of Fujian College of Forestry (Chinese)* **32**, 321-325 (2012).	Soil N:P	2, 3	15
Wang, Q. K. *et al.* Ecosystem carbon storage and soil organic carbon stability in pure and mixed stands of Cunninghamia lanceolata and Michelia macclurei. *Plant Soil* **370**, 295-304 (2013).	Soil C:N	2	28
Wen, L. *et al.* Soil microbial biomass carbon and nitrogen in pure and mixed stands of Pinus massoniana and Cinnamomum camphora differing in stand age. *Forest Ecol. Manag.* **328**, 150-158 (2014).	Soil C:N, MBC/MBN	2	10, 24, 45
Wu, W. *et al.* Coniferous-Broadleaf Mixture Increases Soil Microbial Biomass and Functions Accompanied by Improved Stand Biomass and Litter Production in Subtropical China. *Forests* **10**, 879 (2019).	Soil C:N, N:P, C:P, MBC/MBN, Enzymatic C:N, C:P, N:P	2	34

Xia, Z.-C. *et al.* A broadleaf species enhances an autotoxic conifers growth through belowground chemical interactions. *Ecology* **97**, 2283-2292 (2016).	Soil N:P	2	23
Yamamura, T. *et al.* Tree species identity has little impact on the structure of soil bacterial communities in a 10-year-old tropical tree plantation. *Biol. Fertil. Soils* **49**, 819-828 (2013).	Soil C:N	3, 6	10
Yan, W. D. *et al.* Impacts of changed litter inputs on soil CO_2 efflux in three forest types in central south China. *Chinese Sci. Bull* **58**, 750-757 (2013).	Soil C:N	2	27.5
Yang, J., Xiao, D. & Wan, C. Mixed Experiment of Pinus elliottii and Liquidambar formosana and Analysis of Soil Benefit. *Forest Inventory and Planning* 33, 136 - 138 (2008).	Enzymatic C:N, C:P, N:P	2	10
Yang, Z. *et al.* Litter fall production and carbon return in Cunninghamia lanceolata, Schima superba, and their mixed plantations. *Chinese Journal of Applied Ecology* **21**, 2235-2240 (2010).	Soil N:P	2	20
Zagatto, M. R. G. *et al.* Interactions between mesofauna, microbiological and chemical soil attributes in pure and intercropped Eucalyptus grandis and Acacia mangium plantations. *Forest Ecol. Manag.* **433**, 240-247 (2019).	Soil C:N	2	6
Zeugin, F. *et al.* Is tree diversity an important driver for phosphorus and nitrogen acquisition of a young tropical plantation? *Forest Ecol. Manag.* **260**, 1424-1433 (2010).	Plant N:P	3, 6	6
Zhang, J. *et al.* Content and seasonal change in soil labile organic carbon under different forest covers. *Chinese Journal of Eco-Agriculture* **17**, 41-47 (2009).	Soil C:N	2	24
Zhang, L. *et al.* Nitrogen and phosphorus concentrations, N:P ratio and resorption efficiency of leaves in different forest types. *Journal of Beijing Forestry University* **31**, 67-72 (2009).	Plant N:P	2	11
Zhang, S. *et al.* A Study on Microbial Biomass C, N Characteristics in Different Rehabilitating Forests on Degraded Red Soil. *Acta Agriculture Univesity Jiangxiensis* **32**, 000101-000107 (2010).	Soil C:N, C:P, N:P, MBC/MBN	2, 3	17
Zhang, X. *et al.* Tree species mixture inhibits soil organic carbon mineralization accompanied by decreased r-selected bacteria. *Plant Soil* **431**, 203-216 (2018).	Soil C:N, C:P, N:P	2	31
Zhao, L. & Wang, J. Research on Relations Between Growth Effect and Soil Enzyme Activities and Soil Nutrient Factors in Mixed Poplar and Black Locust Plantations. *Journal of Beijing Forestry University* 17, 1-8 (1995).	Soil C:N, Enzymatic C:N, C:P, N:P	2	10
Zhao, R. D. *et al.* Effects of Plantation Restoration Approaches on Soil Enzyme Activities and Microbial Properties in Hilly Red Soil Region. *Soils* **44**, 576-580 (2012).	Soil C:N, N:P, MBC/MBN, Enzymatic C:N	2	13
Zhou, J. *et al.* Effects of Cunninghamia lanceolata-Betula luminifera Mixed Forests on Soil Microbial Biomass and	MBC/MBN, Enzymatic	2	6

Enzyme Activity. *Journal of Northwest A&F University* **43**, 83-86 (2015).	C:N, C:P, N:P		
**Grassland**			
Abbas, M. et al. Biodiversity Effects on Plant Stoichiometry. Plos One, 8, e58179 (2013).	Plant C:N, C:P, N:P	2, 4, 8, 16	0.5, 1, 2, 3, 4, 5, 6, 7, 8, 9, 10
Bao, X. *et al.* Soil Fertility Improvement by M ixed Planting of Leguminous and Gramineous Green M anure Crops. *Chinese Journal of Grasslan* **34**, 43 - 47 (2012).	Soil N:P	2	2
Chen, D. *et al.* Response of Soil Microbial Biomass C and N, C Metabolism Characteristics of Microbes to Grass-Legume Mixtures of Annual Artificial Grassland in Sanjiangyuan Region. *ACTA AGRESTIA SINICA* **26**, 1644-1670 (2018).	Soil C:N, MBC/MBN	3	0.5
Chen, G. *Effects of Peer Mixed Cropping Oat and Common Vetch on Forage Yield and Quality and Soil Properties* Master thesis, Inner Mongolia Agricultural University, (2017).	Soil N:P, Enzymatic C:N, C:P, N:P	2	0.25
Chen, H. M. *et al.* Plant species richness negatively affects root decomposition in grasslands. *J. Ecol.* **105**, 209-218 (2017).	Plant C:N	2, 4, 8, 16	11
Cong, W. F. & Eriksen, J. Forbs differentially affect soil microbial community composition and functions in unfertilized ryegrass-red clover leys. *Soil Biol. Biochem.* **121**, 87-94 (2018).	Soil C:N	2	3
Cong, W. F. *et al.* Plant species richness leaves a legacy of enhanced root litter-induced decomposition in soil. *Soil Biol. Biochem.* **80**, 341-348 (2015).	Soil C:N	2	12
Creme, A. *et al.* Effects of grasses and a legume grown in monoculture or mixture on soil organic matter and phosphorus forms. *Plant Soil* **402**, 117-128 (2016).	Plant C:N, C:P, N:P, Soil C:N, C:P, N:P, MBC/MBN	2	4.5
Cui, Y. *Effect on production performance and soil properties of artificial grassland mixed sowing oats and common vetc*h Master thesis, Gansu Agriculture University, (2014).	Enzymatic N:P	2	0.4
D'Annibale, A. *et al.* Does introduction of clover in an agricultural grassland affect the food base and functional diversity of Collembola? *Soil Biol. Biochem.* **112**, 165-176 (2017).	Plant C:N, Soil C:N	2	1.17
Frasier, I. *et al.* Effect of different cover crops on C and N cycling in sorghum NT systems. *Sci. Total Environ.* **562**, 628-639 (2016).	Plant C:N, MBC/MBN	2	0.5, 1, 1.5 ,2 ,2.5
Guiz, J. *et al.* Interspecific competition alters leaf stoichiometry in 20 grassland species. *Oikos* **127**, 903-914 (2018).	Plant C:N, C:P, N:P	2, 3, 4, 8	3.5
Gong, J. *et al.* Effect of irrigation on the soil respiration of constructed grasslands in Inner Mongolia, China. *Plant Soil* **395**, 159–172 (2015).	Soil C:N	2	1

Habekost, M. et al. in In: Lange, Markus; Steinbeiss, Sibylle; Habekost, Maike; Gleixner, Gerd; Luo, Guangjuan; Guderle, Marcus; Meyer, Sebastian Tobias (2015): Collection of data on soil carbon (particulate and dissolved) in the Jena Experiment (Main Experiment, time series since 2002). PANGAEA	Soil C:N	2, 4, 8, 16	4
Habekost, M. et al. in In: Lange, Markus; Steinbeiss, Sibylle; Habekost, Maike; Gleixner, Gerd; Luo, Guangjuan; Guderle, Marcus; Meyer, Sebastian Tobias (2015): Collection of data on soil carbon (particulate and dissolved) in the Jena Experiment (Main Experiment, time series since 2002). PANGAEA, https://doi.org/10.1594/PANGAEA.848946	Soil C:N	2, 4, 8, 16	6
Li, H. H. *et al.* Advantages of grass-legume mixture for improvement of crop growth and reducing potential nitrogen loss in a boreal climate. *Agr Food Sci* **28**, 176-189 (2019).	Plant C:N	2	2
Li, Q. *et al.* Grass-legume ratio can change soil carbon and nitrogen storage in a temperate steppe grassland. *Soil Till. Res.* **157**, 23-31 (2016).	Plant C:N, Soil C:N	2	4.5
Li, Q. *et al.* Alfalfa monocultures promote soil organic carbon accumulation to a greater extent than perennial grass monocultures or grass-alfalfa mixtures. *Ecol. Eng.* **131**, 53-62 (2019).	Plant C:N, C:P, N:P	2	4.5
Li, S. *The competitiveness of cocksfoot (Dactylis glomerata L.) with companion and volunteer species under various mixture ratios* Master thesis, Gansu Agriculture University, (2016).	Enzymatic C:N	2	1.5
Liu, M. *et al.* Effects of grass-legume mixtures on the production and photosynthetic capacity of constructed grasslands in Inner Mongolia, China. *Crop Pasture Sci.* **67**, 1188-1198 (2016).	Plant C:N	2	0.75
Mace, O. G., Steinauer, K., Jousset, A., Eisenhauer, N. & Scheu, S. Flood-Induced Changes in Soil Microbial Functions as Modified by Plant Diversity. *Plos One* 11, doi:ARTN e0166349	Enzymatic C:N, C:P, N:P	2, 4, 8, 16	11
Prommer, J. *et al.* Increased microbial growth, biomass, and turnover drive soil organic carbon accumulation at higher plant diversity. *Glob. Chang. Biol.* **26**, 669-681 (2020).	Plant C:N	2, 4, 8, 16	13
Qu, J. *Effects of Intercropping Oat and Common Vetch on Forage Yield and Ouality and Soil Characteristics* Master thesis, InnerMongolia Agricultural University (2017).	Soil N:P, MBC/MBN, Enzymatic C:N, C:P, N:P	2	1.25
Steinbeiss, S. et al. in In: Lange, Markus; Steinbeiss, Sibylle; Habekost, Maike; Gleixner, Gerd; Luo, Guangjuan; Guderle, Marcus; Meyer, Sebastian Tobias (2015): Collection of data on soil carbon (particulate and dissolved) in the Jena Experiment (Main Experiment, time series since 2002). PANGAEA.	Soil C:N	2, 4, 8, 16	2

Sun, Y. M. et al. Influence of intercropping and intercropping plus rhizobial inoculation on microbial activity and community composition in rhizosphere of alfalfa (Medicago sativa L.) and Siberian wild rye (Elymus sibiricus L.). *Fems Microbiology Ecology* 70, 218-226 (2009).	Enzymatic C:N, C:P, N:P	2	1.3
Tai, J. *et al.* Effects of manual sowing methods of Medicago sativa and Bromus inermis on the distribution and content of soil organic carbon and nitrogen. *Acta Prataculturae Sinica* **19**, 41 - 45 (2010).	Soil C:N	2	1
Tang, X. *Effects of Water and Nitrogen Coupling on Pasture Establishment in HulunBuir* Master thesis, Chinese Academy of Agricultural Sciences Dissertation, (2018).	Soil C:N, MBC/MBN	2	1.5
Tribouillois, H. *et al.* Cover crop mixtures including legume produce ecosystem services of nitrate capture and green manuring: assessment combining experimentation and modelling. *Plant Soil* **401**, 347-364 (2016).	Plant C:N	2	0.3
van Eekeren, N. *et al.* Effect of individual grass species and grass species mixtures on soil quality as related to root biomass and grass yield. *Appl. Soil Ecol.* **45**, 275-283 (2010).	Plant C:N, Soil C:N	2	2
van Eekeren, N. *et al.* A mixture of grass and clover combines the positive effects of both plant species on selected soil biota. *Appl. Soil Ecol.* **42**, 254-263 (2009).	Plant C:N, Soil C:N	2	1.75
Wan, Z. *Studies on response of productivity and nitrogen allocation of artificial grassland to precipitation in typical steppe in Inner Mongolia* PhD thesis, Inner Mongolia University (2018).	Plant C:N, Soil C:N	2	2.33
Wei, Y. *Study on Cultivation and Utilization of The Mixture Cropping of Chinese Milk Vetch with Rape and Ryegrass* Master thesis, Huazhong Agricultural University (2013).	Plant C:N, C:P, N:P	2	0.5
Xu, M. et al. Responses of Soil Organic Carbon Fractionation and Microbial Community to Nitrogen and Water Addition in Artificial Grassland. *Scientia Agricultura Sinica* 53, 2678 - 2690 (2020).	Soil C:N, Enzymatic C:N, C:P, N:P	2	3
Zhang, H. *Soil Microbial Diversity under Different Vegetation Restoration Patterns for Hulunbeier Sandy Land* Master thesis, Tianjing Normal University, (2012).	Soil N:P, Enzymatic N:P	2	4
Zhang, Y. *et al.* Response of Ecological Stoichiometry Characteristics of Carbon, Nitrogen and Phosphorus in Root of Forage Plants to Alfalfa-grass Mixture Methods. *Chinese Journal of Grassland* **40**, 59-66 (2018).	Plant C:N, C:P, N:P	2	0.375
**Pot**			
Beyer, F. *et al.* Fine root morphological and functional traits in Fagus sylvatica and Fraxinus excelsior saplings as dependent on species, root order and competition. *Plant Soil* **373**, 143-156 (2013).	Plant C:N	2	3
Chen, J. et al. Shifts in soil microbial community, soil enzymes and crop yield under peanut/maize intercropping with reduced nitrogen levels. *Applied Soil Ecology* 124, 327-334 (2018).	Soil N:P, Enzymatic C:N, C:P, N:P	2	0.17

Chen, M. M. *et al.* Plant growth and soil microbial community structure of legumes and grasses grown in monoculture or mixture. *J. Environ. Sci.-China* **20**, 1231-1237 (2008).	Plant C:N	4	0.2
Fang, S. Z. *et al.* Tree Species Composition Influences Enzyme Activities and Microbial Biomass in the Rhizosphere: A Rhizobox Approach. *Plos One* **8** (2013).	MBC/MBN	2, 3	0.7
Forrester, D. I. *Mixed-species plantations of nitrogen-fixing and non-nitrogen-fixing trees* Doctor of Philosophy thesis, The Australian National University, (2004).	Plant N:P, Soil N:P	2	0.9
Guo, Q. X. *et al.* Species-specific competition and N fertilization regulate non-structural carbohydrate contents in two Larix species. *Forest Ecol. Manag.* **364**, 60-69 (2016).	Plant C:N	2	0.8
Hajek, P. *et al.* Root order- and root age-dependent response of two poplar species to belowground competition. *Plant Soil* **377**, 337-355 (2014).	Plant C:N	2	0.375
Hokka, V. *et al.* Interactive effects of defoliation and an AM fungus on plants and soil organisms in experimental legume–grass communities. *Oikos* **106**, 73-84 (2004).	Plant C:N	2	0.25
Li, Q.-s. et al. Biochemical and microbial properties of rhizospheres under maize/peanut intercropping. *Journal of Integrative Agriculture* 15, 101-110 (2016).	Soil N:P, Enzymatic C:N, C:P, N:P	2	0.17
Sanaullah, M., Blagodatskaya, E., Chabbi, A., Rumpel, C. & Kuzyakov, Y. Drought effects on microbial biomass and enzyme activities in the rhizosphere of grasses depend on plant community composition. *Applied Soil Ecology* 48, 38-44 (2011).	Enzymatic C:N	3	0.2
Scalise, A. *et al.* Pea cultivar and wheat residues affect carbon/nitrogen dynamics in pea-triticale intercropping: A microcosms approach. *Sci. Total Environ.* **592**, 436-450 (2017).	MBC/MBN	2	2
Sun, C. Response Of Rhizospheric Microbiology To Plant Competition And Moisture Stress PhD thesis, Northwest A&F University, (2017).	Plant C:N, N:P, C:P, Enzymatic C:N, C:P, N:P	2, 3	0.33
Teng, W., Liu, S., Cao, F. & Wang, G. Study on chemical and biological properties of potting soil under Camellia oleifera - soybean intercropping. *Journal of Central South University of Forestry & Technology* 33, 24 - 27 (2013).	Soil N:P, Enzymatic N:P	2	0.75
Wang, M. *et al.* Interspecific plant competition increases soil labile organic carbon and nitrogen contents. *Forest Ecol. Manag.* **462**, 117991 (2020).	Plant C:N, Soil C:N	2	1
Yan, M.-c. *et al.* Effects of Different Cropping Patterns of Soybean and Maize Seedlings on Soil Enzyme Activities and MBC and MBN. *Journal of Northeast Agricultural University* **19**, 42-47 (2012).	MBC/MBN	2	0.06
Zhang, C. et al. Effects of rhizosphere interactions of grass interspecies on the soil microbial properties during the	MBC/MBN, Enzymatic	2	0.5

natural succession in the Loess Plateau. *European Journal of Soil Biology* 85, 79-88 (2018).	C:N, C:P, N:P		
Zhang, C., Wang, J., Liu, G. B., Song, Z. L. & Fang, L. C. Impact of soil leachate on microbial biomass and diversity affected by plant diversity. *Plant Soil* 439, 505-523 (2019).	Enzymatic C:N, C:P, N:P	2, 3	0.5

Supplementary Table 4. Effects of functional diversity in mixtures (FD_{is}), background soil nutrient availability (soil C:N, N:P, and C:P ratios) and aridity index on the natural log response ratios (ln*RRs*) of the C:N:P ratios of plant, soil, soil microbial biomass and enzyme across all studies without pot studies.

Source	Estimate	df	F	*P*	R^2	VIF
**Plant C:N ($R^2_{marginal}$ = 0.004, $R^2_{conditional}$= 0.207)**						
FD_{is}	0.006	1,59	0.08	0.773	< 0.001	1.01
log(Background soil C:N)	-0.064	1,42	0.67	0.416	0.004	1.01
**Plant C:P ($R^2_{marginal}$ = 0.261, $R^2_{conditional}$= 0.294)**						
FD_{is}	-0.023	1,15	0.07	0.790	0.002	1.58
Background soil C:P	-0.005	1,16	8.06	**0.012**	0.259	1.58
**Plant N:P ($R^2_{marginal}$ = 0.004, $R^2_{conditional}$= 0.200)**						
FD_{is}	0.038	1,64	0.55	0.463	0.004	
**Soil C:N ($R^2_{marginal}$ = 0.083, $R^2_{conditional}$= 0.512)**						
FD_{is}	0.015	1,348	0.29	0.590	< 0.001	1.01
Background soil C:N	-0.010	1,217	51.20	**<0.001**	0.083	1.01
**Soil C:P ($R^2_{marginal}$ = 0.197, $R^2_{conditional}$= 0.626)**						
FD_{is}	0.158	1,43	1.85	0.181	0.019	1.00
Background soil C:P	-0.002	1,56	17.73	**<0.001**	0.178	1.00
**Soil N:P ($R^2_{marginal}$ = 0.118, $R^2_{conditional}$= 0.343)**						
FD_{is}	0.044	1,125	0.41	0.523	0.003	1.00
Background soil N:P	-0.033	1,63	17.37	**<0.001**	0.115	1.00
**Microbial biomass C:N ($R^2_{marginal}$ = 0.045, $R^2_{conditional}$= 0.273)**						
FD_{is}	-0.256	1,125	6.45	**0.012**	0.020	1.41
AI	-0.309	1,112	8.13	**0.005**	0.025	1.41
**Enzyme C:N ($R^2_{marginal}$ = 0.012, $R^2_{conditional}$= 0.282)**						
FD_{is}	0.148	1,191	3.68	0.057	0.012	
**Enzyme C:P ($R^2_{marginal}$ = 0.031, $R^2_{conditional}$= 0.149)**						
log(FD_{is})	0.129	1,64	6.66	**0.012**	0.031	
**Enzyme N:P ($R^2_{marginal}$ < 0.001, $R^2_{conditional}$= 0.264)**						
FD_{is}	0.026	1,162	0.150	0.699	<0.001	

Note: Linear mixed-effects model fit tests used Satterthwaite approximations for denominator degrees of freedom (df). *P* and R^2 are the significance of the model and explained variance by the model, respectively.

Supplementary Table 5. Values of Akaike information criterion for the linear and log-linear functional diversity (*FD*is), stand age (S*A*), and background nutrient availability (*N*).

Attribute	FD_{is}	$\ln(FD_{is})$	SA	ln(SA)	N	ln(N)
Plant C:N	**-328.1**	-328.8	**-328.0**	-327.6	-261.6	**-263.9**
Plant C:P	**-209.8**	-210.5	**-210.2**	-210	**-111.5**	-112.6
Plant N:P	**-128.1**	-128.2	**-127.9**	-128	**-20.3**	-17.5
Soil C:N	**-616.2**	-615.5	**-623.7**	-618.5	**-663.5**	-658.5
Soil C:P	**48.6**	49	**48.2**	49	**37.6**	45.2
Soil N:P	**105.3**	105.2	**100.3**	104	**87.5**	86.6
Microbial C:N	**117.4**	117	**116.8**	118.3	**81.1**	81
Enzymatic activities C:N	**147.5**	145.7	**150.2**	150.6	**22.5**	22.2
Enzymatic activities C:P	101.8	**99.5**	**102.9**	103.5	**11.0**	10.6
Enzymatic activities N:P	**77.7**	77.6	**76.0**	76.2	**20.9**	20.7

Supplementary Table 6. Results from the Egger's tests of publication bias on our findings. We used funnel tests of asymmetry with the sample size as the predictor. In all cases, we analyzed the response ratio across the entire dataset (denoted by the C:N:P ratio variable label) followed by the significant covariate models that we present in the method (Eqns. 5, 6, 7). P-values < 0.05 indicates a significant publication bias.

	z	*p*
Plant C:N	0.36	0.714
+covariates	0.42	0.672
Plant C:P	0.10	0.922
+covariates	0.03	0.975
Plant N:P	-0.36	0.722
+covariates	-0.32	0.747
Soil C:N	0.06	0.950
+covariates	0.14	0.885
Soil C:P	-0.92	0.359
+covariates	-0.83	0.407
Soil N:P	-0.25	0.801
+covariates	-0.25	0.804
Soil microbial biomass C:N	-0.38	0.704
+covariates	-0.34	0.737
Soil enzyme C:N	-0.23	0.816
+covariates	-0.11	0.911
Soil enzyme C:P	-0.33	0.743
+covariates	-0.20	0.843
Soil enzyme N:P	-0.49	0.627
+covariates	-0.43	0.668

a

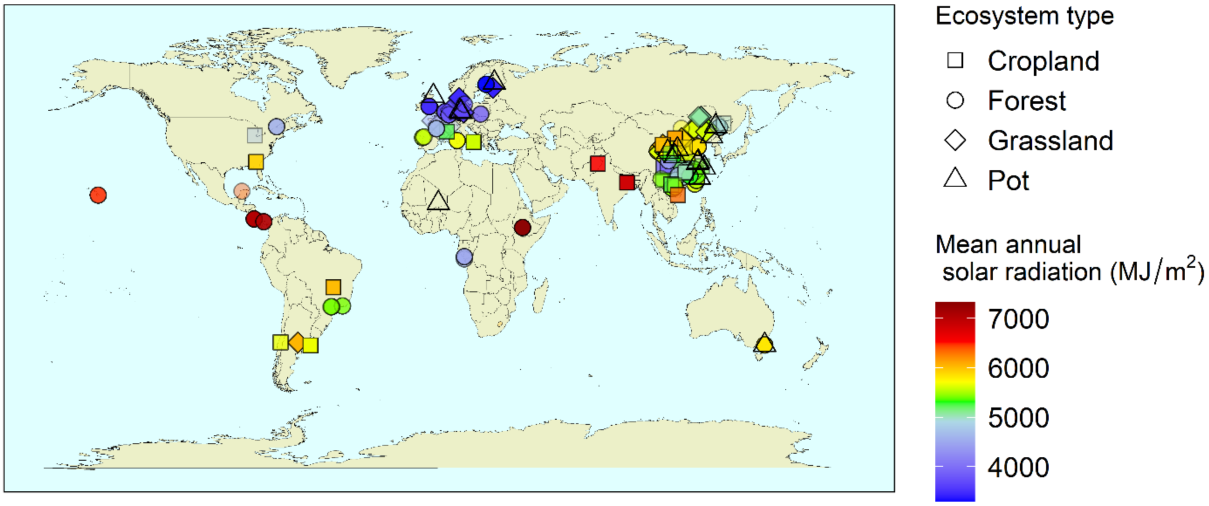

Ecosystem type
Cropland
Forest
Grassland
Pot
Mean annual
solar radiation (MJ/m2)
7000
6000
5000
4000

b

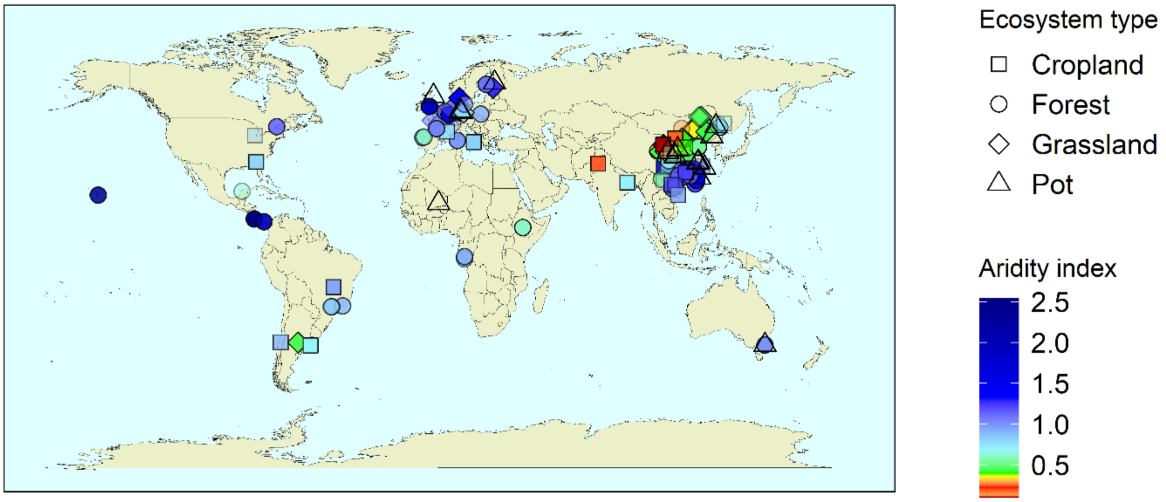

Ecosystem type
Cropland
Forest
Grassland
Pot
Aridity index
2.5
2.0
1.5
1.0
0.5

38

Supplementary Figure 1. Global distribution of plant diversity experiments testing the effects of plant diversity on terrestrial C:N:P ratio stoichiometry collected for this meta-analysis. Square, circle, rhombus and triangle points indicate study sites at croplands, forests, grasslands, and pots. Aridity index indicates more climate moisture availability.

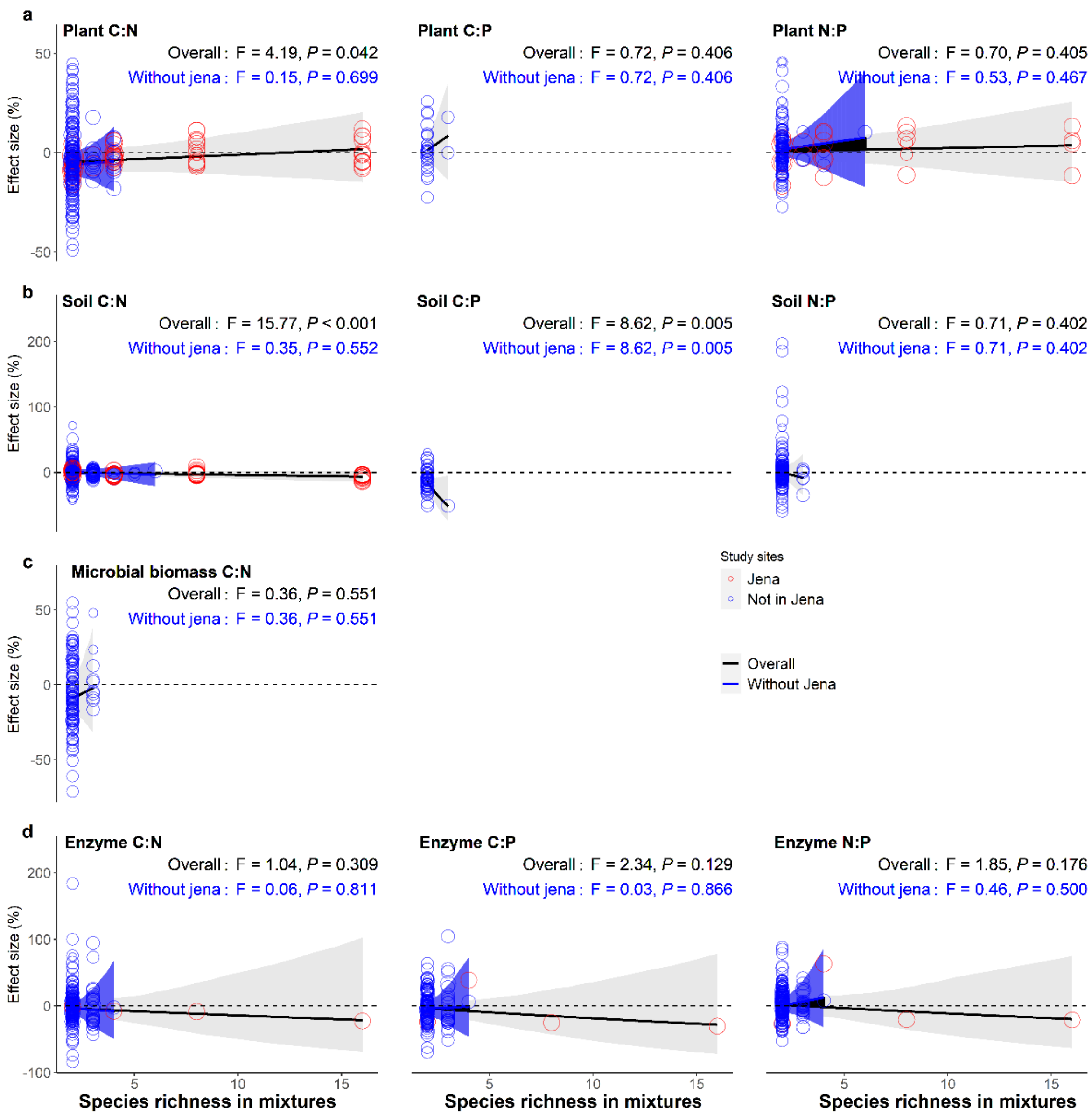

Supplementary Figure 2. Comparison of C:N:P ratios of plant, soil, soil microbial biomass and enzyme in species mixtures versus monocultures in relation to the species richness in mixtures for the datasets with and without Jena experiment. a, plant C:N, C:P and N:P ratios; **b,** soil C:N, C:P and N:P ratios; **c,** soil microbial C:N ratio; **d,** soil enzyme C:N, C:P and N:P ratios. The effects are quantified as the percent changes in mixtures compared to the corresponding mean value of constituent monocultures. Points represent the values predicted by partial regressions for each explanatory variable, with their sizes representing the relative

weights of corresponding observations. Red and blue points indicate study sites at Jena and not at Jena. Black lines and grey shade areas respectively represent the average responses to species richness and their bootstrapped 95% confidence intervals in datasets with Jena experiment. Blue lines and blue shade areas respectively represent the average responses to species richness and their bootstrapped 95% confidence intervals in datasets without Jena experiment. Slope estimates are partial dependence, derived from the full model (see Methods). We found that species richness and functional diversity yielded qualitatively similar trends for all C:N:P variables, except soil C:N and C:P, which significantly decreased with species richness. But these results are driven by a single study (Jena) with high species richness (4, 8, 16) or limited species richness levels for some selected variables (2 levels soil C:P), respectively.

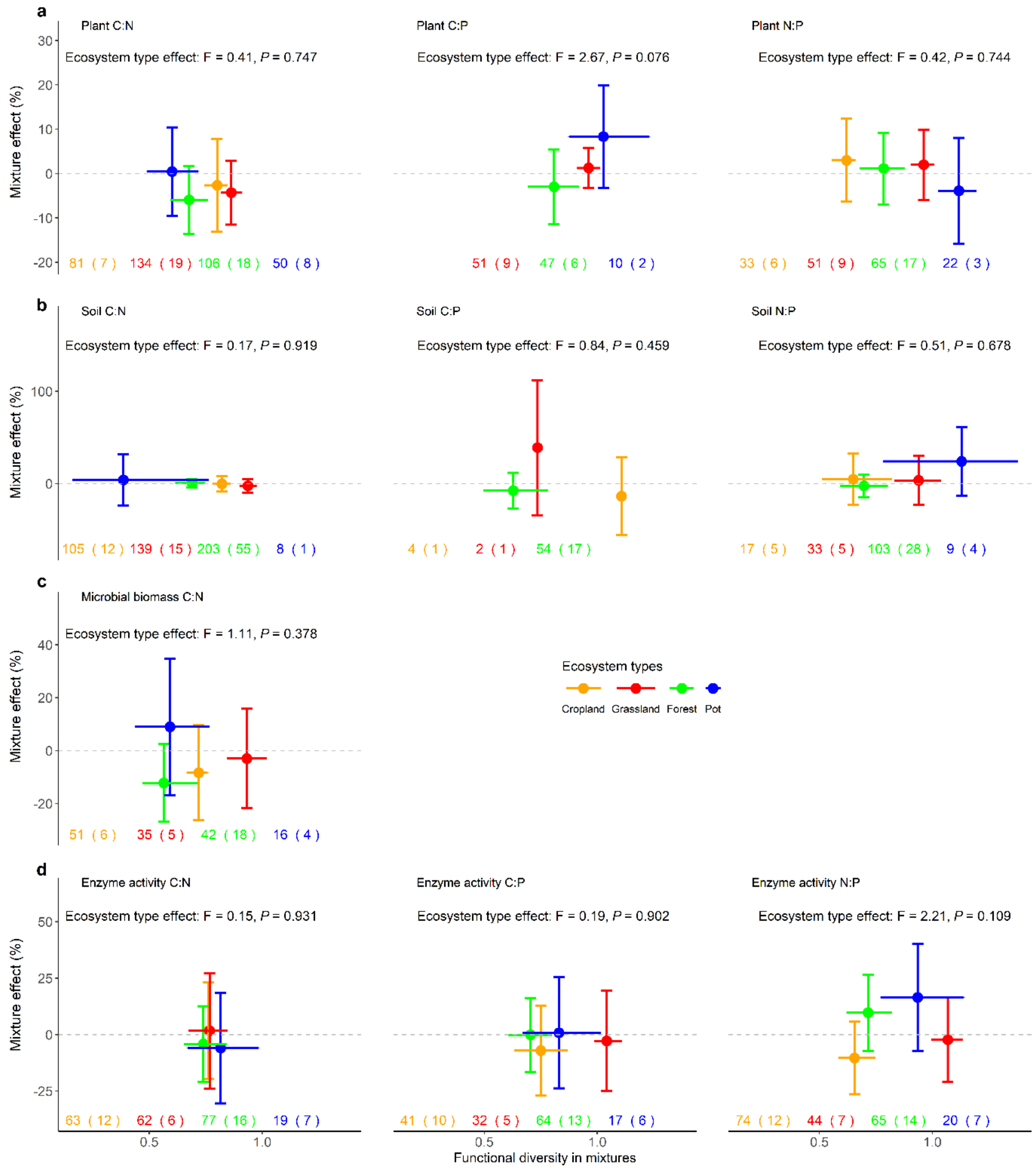

Supplementary Figure 3. Comparison of C:N:P ratios of plants, soils, soil microbial biomass and enzymes in species mixtures versus monocultures between the four ecosystem types. Means and vertical and horizontal error bars represent means and 95% confidence intervals for species mixture effects and the functional diversity in mixtures, respectively. Orange, red, green and bule points and error bars indicate study sites at croplands, grasslands,

forests, and pots. For each experimental system, the number of observations is shown, with the number of studies in parentheses. P values, derived from the linear mixed model with the experimental system as the fixed effect and study as the random effect, represent the significance of the difference in the natural log response ratios (ln*RR*s) between experimental systems. Despite significant differences in the species richness in mixtures between experimental systems (as indicated by non-overlapping CIs), ln*RR* did not differ significantly for any of the attributes studied.

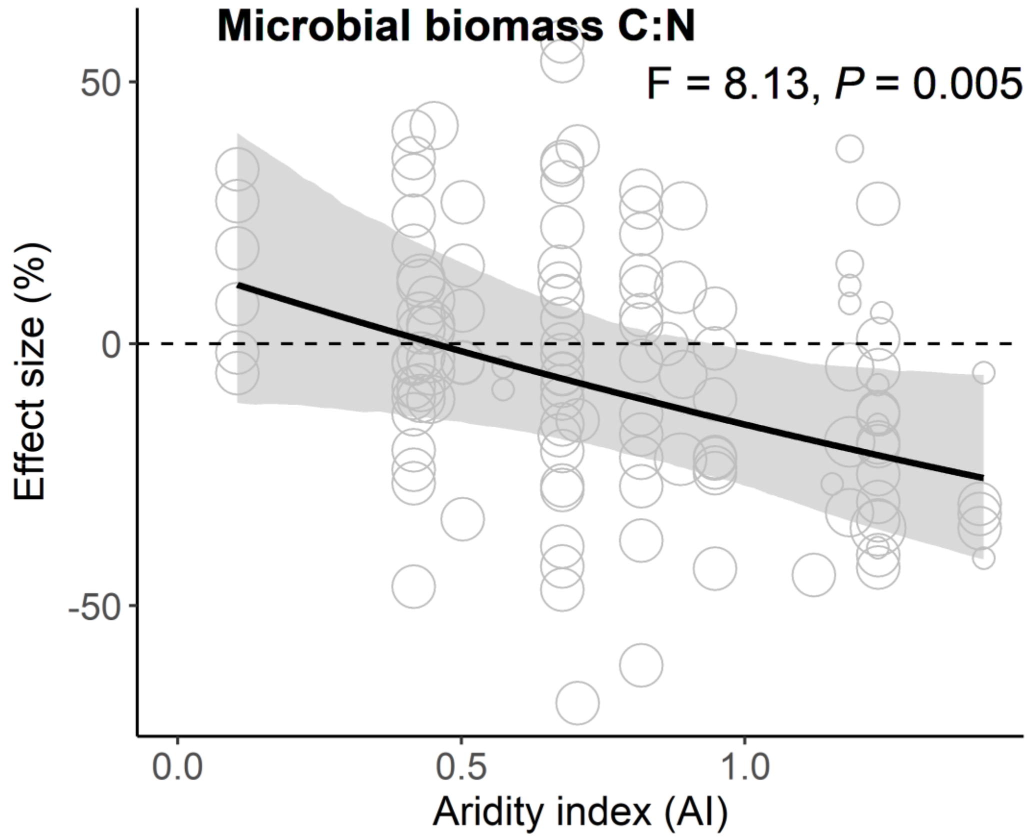

Supplementary Figure 4. Comparison of soil microbial C:N ratio in species mixtures versus monocultures in relation to aridity index. The effects are quantified as the percent changes in mixtures compared to the corresponding mean value of constituent monocultures. Points represent the values predicted by partial regressions for each explanatory variable, with their sizes representing the relative weights of corresponding observations. Slope estimates are partial dependence, derived from the full model (see Methods). Black lines represent the average responses with their bootstrapped 95% confidence intervals shaded in grey. Aridity index indicates more climate moisture availability.

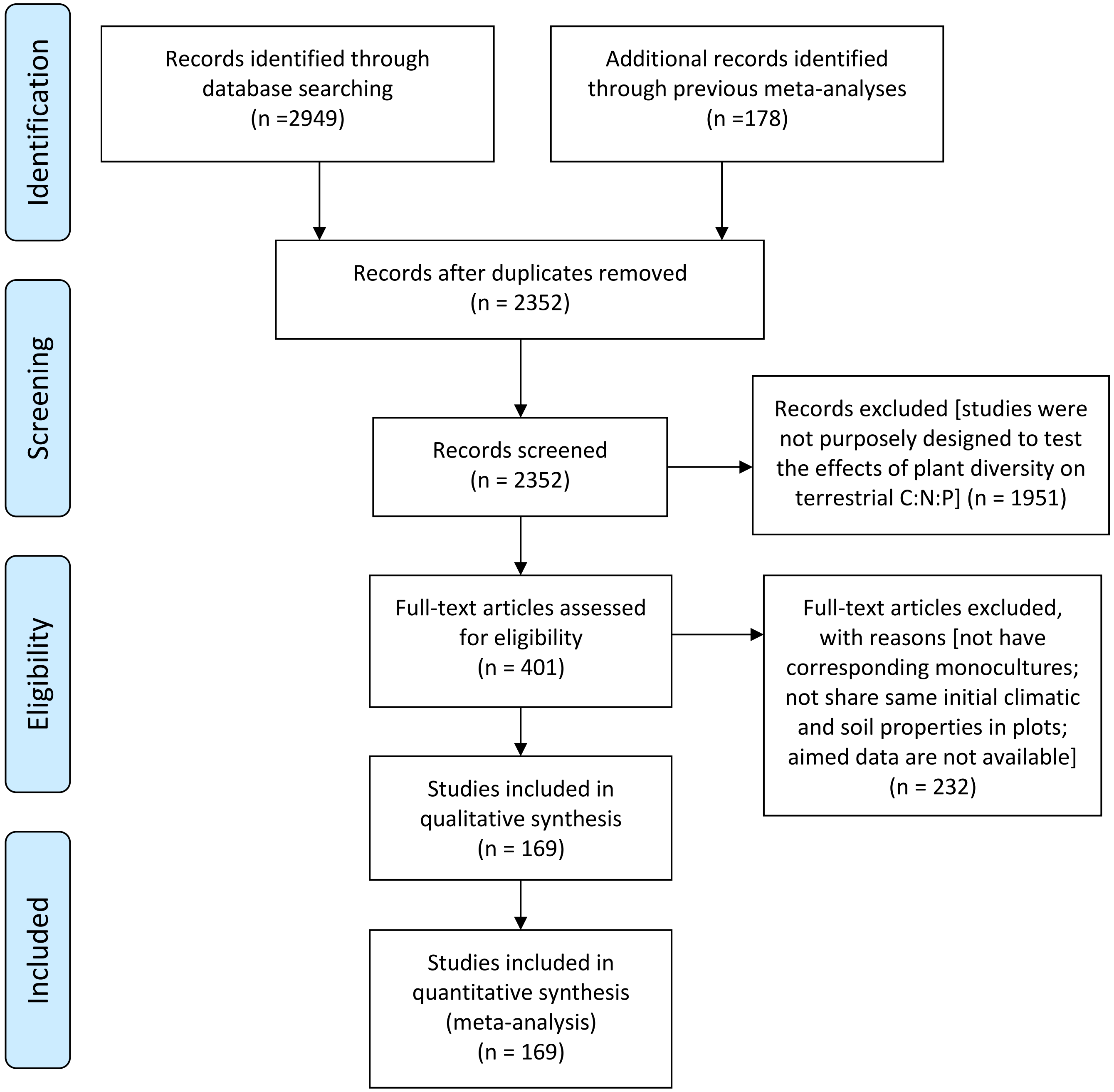

Supplementary Figure 5. PRISMA diagram showing the process for locating publications included in this meta-analysis.
