# Supplementary material for: Plant mixture balances terrestrial ecosystem C:N:P stoichiometry
Source: Nat Commun. 2021 Jul 27;12:4562. doi: 10.1038/s41467-021-24889-w (PMC8316448; doi:10.1038/s41467-021-24889-w)
Supplement: Supplementary file 3 — Reporting Summary [file 41467_2021_24889_MOESM3_ESM.pdf]

nature portfolio

Corresponding author(s): Han Y. H. Chen

Last updated by author(s): Jul 13, 2021

Reporting Summary

Nature Portfolio wishes to improve the reproducibility of the work that we publish. This form provides structure for consistency and transparency in reporting. For further information on Nature Portfolio policies, see our Editorial Policies and the Editorial Policy Checklist.

Statistics

For all statistical analyses, confirm that the following items are present in the figure legend, table legend, main text, or Methods section.

n/a	Confirmed	
☐	☒	The exact sample size (*n*) for each experimental group/condition, given as a discrete number and unit of measurement
☐	☒	A statement on whether measurements were taken from distinct samples or whether the same sample was measured repeatedly
☐	☒	The statistical test(s) used AND whether they are one- or two-sided<br>*Only common tests should be described solely by name; describe more complex techniques in the Methods section.*
☐	☒	A description of all covariates tested
☐	☒	A description of any assumptions or corrections, such as tests of normality and adjustment for multiple comparisons
☐	☒	A full description of the statistical parameters including central tendency (e.g. means) or other basic estimates (e.g. regression coefficient) AND variation (e.g. standard deviation) or associated estimates of uncertainty (e.g. confidence intervals)
☐	☒	For null hypothesis testing, the test statistic (e.g. *F*, *t*, *r*) with confidence intervals, effect sizes, degrees of freedom and *P* value noted<br>*Give P values as exact values whenever suitable.*
☒	☐	For Bayesian analysis, information on the choice of priors and Markov chain Monte Carlo settings
☐	☒	For hierarchical and complex designs, identification of the appropriate level for tests and full reporting of outcomes
☒	☐	Estimates of effect sizes (e.g. Cohen's *d*, Pearson's *r*), indicating how they were calculated

Our web collection on statistics for biologists contains articles on many of the points above.

Software and code

Policy information about availability of computer code

Data collection	All peer-reviewed publications that were published prior to May 2021, which investigated the effects of plant diversity on terrestrial C:N:P ratios (i.e., plants, soils, soil microbial biomass and enzymes) were collected by searching the Web of Science (Core Collection; http://www.webofknowledge.com), Google Scholar (http://scholar.google.com), and the China National Knowledge Infrastructure (CNKI; https://www.cnki.net) . Plot Digitizer version 2.0 (Department of Physics at the University of South Alabama, Mobile, AL, USA) was used to extract data from the figures. The mean annual solar radiation and aridity index data were retrieved from the CGIAR-CSI Global Aridity Index dataset and WorldClim Version 2 using location information. We obtained the mean trait values of Nmass and SLA data by using all available measurements for each plant species from the TRY Plant Trait Database.
Data analysis	The data analysis was conducted in R 4.0.2 (R Core Team, 2021), using packages including 'data.table (version 1.13.6)', 'lme4 (version 1.1-26)', 'lmerTest (version 3.1-3)', 'ggplot2 (version 3.3.3)', 'maps (version 3.3.0)', 'boot (version 1.3-27)' and 'FD (version 1.0-12)'. Details were reported in statistical analysis section of the Methods.

For manuscripts utilizing custom algorithms or software that are central to the research but not yet described in published literature, software must be made available to editors and reviewers. We strongly encourage code deposition in a community repository (e.g. GitHub). See the Nature Portfolio guidelines for submitting code & software for further information.

Data

Policy information about availability of data

All manuscripts must include a data availability statement. This statement should provide the following information, where applicable:

- Accession codes, unique identifiers, or web links for publicly available datasets
- A description of any restrictions on data availability
- For clinical datasets or third party data, please ensure that the statement adheres to our policy

The source data underlying Figs. 1–4 and Supplementary Figures 1–5 and Supplementary Tables 1–6 have been deposited in Figshare (https://doi.org/10.6084/m9.figshare.13392809.v2).

Field-specific reporting

Please select the one below that is the best fit for your research. If you are not sure, read the appropriate sections before making your selection.

☐ Life sciences ☐ Behavioural & social sciences ☒ Ecological, evolutionary & environmental sciences

For a reference copy of the document with all sections, see nature.com/documents/nr-reporting-summary-flat.pdf

Ecological, evolutionary & environmental sciences study design

All studies must disclose on these points even when the disclosure is negative.

Study description	We conducted a global meta-analysis with 2049 paired observations of plant monocultures and mixtures from 169 studies, to evaluate the responses of plant and soil C:N:P ratios to plant species mixtures. We investigated whether the plant mixture effects on the C:N, C:P, N:P ratios of plants, soils, microbial biomass and enzymes were affected by functional diversity in mixtures and the status of background nutrients.
Research sample	The data for this study was obtained from 169 peer-reviewed publications that investigated the effects of plant diversity on plant and soil C:N:P ratios, using the Web of Science and Google Scholar, up to May 2021. The 169 publications were listed in Supplementary Table 3 .
Sampling strategy	We systematically searched all peer-reviewed publications that were published prior to May 2021, which investigated the effects of plant diversity on terrestrial C:N:P ratios (i.e., plants, soils, soil microbial biomass and extracellular enzymes) using the Web of Science (Core Collection; http://www.webofknowledge.com), Google Scholar (http://scholar.google.com), and the China National Knowledge Infrastructure (CNKI; https://www.cnki.net) using the search term: “C:N or C:P or N:P or C:N:P AND plant OR soil OR microbial biomass OR extracellular enzyme OR exoenzyme AND plant diversity OR richness OR mixture OR pure OR polyculture OR monoculture OR overyielding”, and also searched for references within these papers. Our survey also included studies summarized in previously published diversity-ecosystem functioning meta-analyses. We employed the following criteria to select the studies: (i) they were purposely designed to test the effects of plant diversity on C:N:P ratios, (ii) they had at least one species mixture treatment and corresponding monocultures, (iii) they had the same initial climatic and soil properties in the monoculture and mixture treatment plots. In thirteen publications, several experiments, each with independent controls, were conducted at different locations and were considered to be distinct studies. The literature search was performed following the guidelines of PRISMA (Preferred Reporting Items for Systematic Reviews and Meta-Analyses, Supplementary Fig. 5). In total, 169 studies met these criteria (Supplementary Fig. 5 and Supplementary Table 3). When different publications included the same data, we recorded the data only once. When a study included plant species mixtures of different numbers of species, we considered them as distinct observations. This resulted in 52 studies for plant C:N ratios, 35 studies for plant N:P ratios, 17 studies for plant C:P ratios, 83 studies for soil C:N ratios, 42 studies for soil N:P ratios, 19 studies for soil C:P ratios, 33 studies for soil microbial biomass C:N ratios, 41 studies for soil enzyme C:N ratios, 40 studies for soil enzyme N:P ratios and 34 studies for soil enzyme C:P ratios. The number of studies are sufficient for the meta analysis.
Data collection	Xinli Chen collected the data. For each site, we extracted the means, the number of replications, and standard deviations of the C:N, N:P, and C:P ratios of plants (including leaves, shoots, fine roots, total roots), soils, soil enzymes as well as soil microbial biomass C:N ratios, if reported. When an original study reported the results graphically, we used Plot Digitizer version 2.0 (Department of Physics at the University of South Alabama, Mobile, AL, USA) to extract data from the figures. We also extracted species compositions in mixtures, latitude, longitude, stand age, ecosystem type (i.e, forest, grassland, cropland, pot), mean annual temperature (MAT, °C), management practice (fertilization or not), soil type (FAO classification) and sampled soil depth from original or cited papers, or cited data sources. The mean annual solar radiation and aridity index data were retrieved from the CGIAR-CSI Global Aridity Index dataset and WorldClim Version 2 using location information. The annual aridity index was calculated as the ratio of the mean annual precipitation to mean annual potential evapotranspiration. Stand age (SA) was recorded as the number of years since stand establishment following stand-replacing disturbances in forests, and the number of years between the initiation and measurements of the experiments in grasslands, croplands and pots. The species proportions in plant mixtures were based on the stem density in forests and pots, coverage in croplands, and sown seeds in grasslands. Soil depth was recorded as the midpoint of each soil depth interval. We employed the weighted averages of soil C:N, C:P, and N:P ratios of monocultures in each study as proxies for the status of background nutrients. For studies that did not report soil C:N, C:P, and N:P ratios of monocultures, we used the initial soil C:N, C:P, and N:P ratios (before experiment establishment, if reported) as proxies for the status of background nutrients.
Timing and spatial scale	The searching for publication and data collection continuously proceeded from Jan 18th, 2020 to May 2ed, 2021.<br>The timing scale of the observations in original studies ranged from 1999 to 2021.<br>The observations were distributed in global terrestrial ecosystems.

Data exclusions	No data were excluded from the analyses.
Reproducibility	All attempts to repeat the analysis and results were successful.
Randomization	The data were analyzed with a random effect model. Bootstrapping sampling was used.
Blinding	Complete blinding in bootstrapping process.

Did the study involve field work? ☐ Yes ☒ No

Reporting for specific materials, systems and methods

We require information from authors about some types of materials, experimental systems and methods used in many studies. Here, indicate whether each material, system or method listed is relevant to your study. If you are not sure if a list item applies to your research, read the appropriate section before selecting a response.

Materials & experimental systems

n/a	Involved in the study	
☒	☐	Antibodies
☒	☐	Eukaryotic cell lines
☒	☐	Palaeontology and archaeology
☒	☐	Animals and other organisms
☒	☐	Human research participants
☒	☐	Clinical data
☒	☐	Dual use research of concern

Methods

n/a	Involved in the study	
☒	☐	ChIP-seq
☒	☐	Flow cytometry
☒	☐	MRI-based neuroimaging
